# Soil biodiversity and function under global change

**DOI:** 10.1371/journal.pbio.3003093

**Published:** 2025-03-27

**Authors:** Manuel Delgado-Baquerizo, David J. Eldridge, Yu-Rong Liu, Zhong-Wen Liu, Claudia Coleine, Pankaj Trivedi

**Affiliations:** 1 Laboratorio de Biodiversidad y Funcionamiento Ecosistémico, Instituto de Recursos Naturales y Agrobiología de Sevilla (IRNAS), Consejo Superior de Investigaciones Científicas (CSIC), Sevilla, Spain; 2 Centre for Ecosystem Science, School of Biological, Earth and Environmental Sciences, University of New South Wales, Sydney, Australia; 3 State Key Laboratory of Agricultural Microbiology and College of Resources and Environment, Huazhong Agricultural University, Wuhan, China; 4 Department of Ecological and Biological Sciences, University of Tuscia, Viterbo, Italy; 5 Microbiome Network and Department of Agricultural Biology, Colorado State University, Fort Collins, Colorado, United States of America; 6 Department of Plant and Soil Science, Institute of Genomics for Crop Abiotic Stress Tolerance (IGCAST), Texas Tech University, Lubbock, Texas, United States of America

## Abstract

Soil organisms represent the most abundant and diverse organisms on the planet and support almost every ecosystem function we know, and thus impact our daily lives. Some of these impacts have been well-documented, such as the role of soil organisms in regulating soil fertility and carbon sequestration; processes that have direct implications for essential ecosystem services including food security and climate change mitigation. Moreover, soil biodiversity also plays a critical role in supporting other aspects from One Health—the combined health of humans, animals, and the environment—to the conservation of historic structures such as monuments. Unfortunately, soil biodiversity is also highly vulnerable to a growing number of stressors associated with global environmental change. Understanding how and when soil biodiversity supports these functions, and how it will adapt to changing environmental conditions, is crucial for conserving soils and maintaining soil processes for future generations. In this Essay, we discuss the fundamental importance of soil biodiversity for supporting multiple ecosystem services and One Health, and further highlight essential knowledge gaps that need to be addressed to conserve soil biodiversity for the next generations.

## Abstract 2

Soil biodiversity is critical for supporting the health of humans, animals and the environment. However, global change threatens our soil ecosystems. This Essay discusses the importance of soil biodiversity in the face of global stressors, and the major knowledge gaps precluding its conservation for future generations.

## Soils: A non-renewable resource critical for human well-being

**Fig 1:**
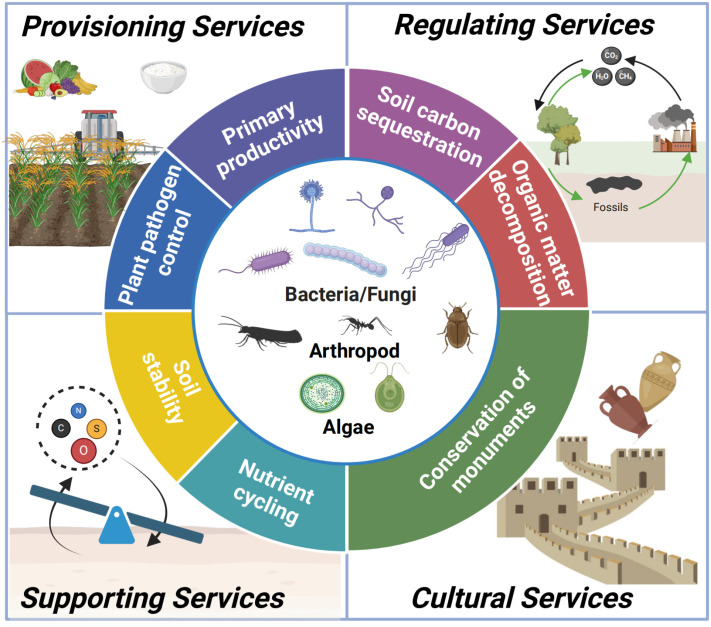
Soil biodiversity, functions, and ecosystem services. Soil biodiversity supports a myriad of ecosystem services associated with multiple aspects, from carbon sequestration and pathogen control to monument conservation.

Soils are fundamental for human survival [1]. Soils support 95% of all the food that we consume [2], sequester three times more carbon than the atmosphere and vegetation [3], and constitute one of the largest reservoirs of human, animal, and plant pathogens [4], as well as antibiotic resistance genes [5]. Because of this, soils are also critical to support the combined and interconnected health of people, animals, and ecosystems under global change (One Health) [5]. Unfortunately, a third of all soils are already significantly degraded. In the case of the European Union, it has been estimated that less than 40% of all soils are in a healthy condition [6]. The problem is that soil is a non-renewable resource, and a single centimeter of soil can take up to thousands of years to develop [7], particularly in ecosystems of low productivity such as those from arid and cold environments. Learning how to conserve and restore our soils is a major priority in many regions of the world, yet we are still far from being able to understand how to preserve our soils for future generations. Understanding the soil ecosystem, including the biodiversity of our soils and its interactions with the environment, is the first step toward ensuring the conservation of our soils. It has recently been estimated that 6 out of 10 species depend directly on soil for their survival [8]. This includes vascular and non-vascular (e.g., mosses) plants, but also a myriad of soil organisms ranging from microbes (viruses, archaea, bacteria, fungi, and protists) to biocrusts (e.g., lichens) and soil micro- and macrofauna (e.g., mammals, nematodes, collembola, earthworms, ants and termites). Further, soil organisms are fundamentally important for supporting multiple ecosystem functions (i.e., multifunctionality) such as soil carbon sequestration, organic matter decomposition, plant pathogen control, soil stability, nutrient cycling, or primary productivity, to name a few (Fig 1) [9,10]. These functions are critical for supporting ecosystem services such as soil fertility, food production, or climate change mitigation, while also contributing to key United Nation Sustainable Development Goals such as Zero Hunger and Climate Action, among others.

## Soil biodiversity and function

The immensity of soil biodiversity is difficult to visualize [11,12]. As an example of this, nematodes are the most abundant animals on the planet, accounting for 80% of all animals worldwide [11]. Also, a single gram of soil, the amount of soil that can be held in a teaspoon, supports thousands of species, and millions of individuals of bacteria [12]. This teaspoon of soil already supports more individuals of bacteria than the number of people on the planet [12]. Strikingly, despite the immense abundance and number of taxa of soil microorganisms (e.g., bacteria, fungi, viruses, and protists), we also know that microbial communities are dominated by a few largely abundant soil taxa (e.g., bacteria), which represent the largest portion of soil microbial populations [13]. Understanding this microbiome is imperative to conserving and restoring our soils. The rapid development of “-omics” techniques in the early 2000s turned the soil microbiome upside down and spurred a resurgence of interest and knowledge of the drivers and functional capabilities of soil microbes [12,14]. Today, we know, for example, that soil biodiversity (the number of “species” of bacteria, fungi, protists, and invertebrates) is often positively associated with soil multifunctionality and stability in natural, urban, and agricultural ecosystems [9,10,12,15–17]. Soil biodiversity was traditionally considered to be highly functionally redundant, meaning that the loss of certain soil taxa was thought to have little impact on soil functions because the functions of these lost taxa would be replaced by others. However, most recent research suggests that losses in microbial diversity can result in proportional or exponential (e.g., in the case of specialized processes such as denitrification), losses of soil function [9,10,17]. This is particularly obvious in extremely complex, comprehensive soil processes such as organic matter decomposition, which require the cooperation of multiple organisms bringing together multiple tools to tackle the large and complex number of metabolic routes involved in this process [17,18]. The decomposition of organic matter and soil pollutants is one of the most important ecosystem services provided by soil organisms on our planet, supporting the release of nutrients, which are then used by plants and soil organisms to grow, further contributing to carbon sequestration. Of course, we also know that not all biodiversity has the same importance in relation to supporting multifunctionality. For example, soil biodiversity has been shown to be particularly critical for supporting function in drylands (which cover almost half of the planet), and poorly developed soils [19]. These poor soils have a small stock of nutrients and organic matter, and thus, the entrance of nutrients depends on the daily contribution of diverse soil microbiota to decompose and depolymerize litter and organic matter. Similarly, it is known that some taxa play a more significant role than others in supporting soil function. Larger organisms (e.g., earthworms, nematodes, Platyhelminthes) are crucial for maintaining soil health by facilitating organic matter decomposition, enhancing nutrient cycling, improving soil aeration, and supporting microbial communities; while smaller organisms (e.g., bacteria, fungi, and protists) are fundamental for supporting a large number of functions, though at lower to medium levels of activity [9]. Much less is known, however, about how viruses influence ecosystem function, although a growing number of studies are tacking this question [20]. Collectively, soil organisms are key drivers of soil function, and their conservation is essential for the sustainability of life on Earth.

Soil organisms also include some major ecosystem engineers, such as small mammals, ants, termites, earthworms, and soil biological crusts (hereafter biocrusts). Recent global-scale research is providing solid evidences of the contributions of these major ecosystem engineers to support soil function [21–23]. Ants and termites are globally distributed social colonial insects that have major effects on soil physical and chemical properties. As central-place foragers, the nest is the center of their activity where food and resources are moved to support colonies of up to 100,000 individual workers. Termite colonies may reach many millions in tropical mound-building species [24]. Global estimates of soil turnover by ants and termites range from 0.001 to 10 t ha^−1^ yr^−1^ [25], and these rates impact soil formation in drylands [26]. Termite and ant nests alter soil’s physical (e.g., texture; [26]) and chemical conditions, mainly by enhancing soil organic matter levels through their foraging, or by transporting subsoil to the surface. This material can be an important source of nutrients for plant growth [21]. In addition, ant nests are known to influence soil microbial communities by promoting fungal and bacterial diversity and represent a reservoir for soil-borne plant pathogens [27].

Another group of key soil engineer are the biocrusts. Biocrusts, communities of small organisms dominated by lichens, bryophytes, algae, cyanobacteria, and fungi, can be found all across the globe. These organisms are thought to occupy about 12% of Earth’s terrestrial land area [28], particularly in areas with low levels of disturbance and reduced plant cover, and have a vast capacity to disperse long distances. Long-distance spore dispersal can occur with wind-blown sediments, or in the case of lichens, fragments that contain both the fungal and algal components on atmospheric dust, and sometimes, on migratory birds. In fact, many biocrusts genera are found right across the globe (e.g., *Psora*, *Diploschistes*). Biocrusts are critical for sustaining a number of essential ecosystem functions and services. Crusts provide a protective shield on the soil surface a few millimeters thick, ensuring that the soil surface is protected against both wind and water erosion [28]. Biocrusts also moderate the flow of water into the soil, but their effect is strongly dependent on soil type, climatic zone, and particular biocrust type [29]. Moreover, a recent global study suggested that biocrusts can support soil carbon stocks, nutrient content, pathogen regulation, and promote microbial activity compared with surrounding bare soils [30]. It is vital that we understand how these soil engineers interact with the other huge range of soil biodiversity to influence soil function. Small soil mammals also act as soil engineers, including globally distributed rabbits and hares, North American prairie dogs (*Cynomys* sp.), and Australian bilbies (*Macrotis lagotis*), which are well-known to dig small burrows and holes, creating new niches for other soil organisms [23].

## Soil biodiversity: From One Health to monument conservation

Remarkably, soil biodiversity is not only important for soil function, but for every single aspect of our life on Earth, from ecosystem restoration and food production to One Health and even the conservation of monuments (Fig 1). For example, microbial bioproducts (e.g., synthetic communities; SynComs) that support a greater diversity of native organisms are often more successful at supporting food production and restoration processes [31]. Harnessing the soil microbiome of croplands to support food production, and understanding the interactions of this crop microbiome with crop species is critical to support food security and agricultural sustainability [32]. Moreover, soil biodiversity is essential for providing a barrier that prevents the entry of pathogens into the soil, and therefore for supporting the health of humans, animals, and the environment, i.e., One Health [5,33,34]. Soils support pathogens such as *Fusarium*, *Alternaria*, or *Phytophthora* threatening food production under climate change [4,34]. Moreover, soils support organisms such as *Mycobacterium*, *Clostridium tetani*, *Yersinia pestis*, or *Aspergillus*, which are of vital importance to human health [5,34]. Recent research suggests that soils that have a greater diversity of native microbes also support a lower proportion of pathogens. The microbiome of healthy soils can be transplanted to fight the spread of pathogens affecting food production [33]. Additionally, soil organisms such as earthworms, which are linked to soil fertility and organic matter decomposition, also provide a cleaning service by removing antibiotic resistance genes from our soils [35].

Soil organisms are also crucial for the conservation of our monuments [36–38]. A recent study, for example, suggested that the microbiome (mainly Actinobacteria) covering a ~ 2,000-year-old tomb from China comprised a subset of the taxa found in the soils outside of this tomb [36]. Similarly, soil mosses have been instrumental in conserving the China Great Wall from air and water erosion [39]. Understanding soil biodiversity is, therefore, not only fundamental for soil processes but also for the multiple aspects surrounding our lives.

## Soil ecological networks and function

Soils are inhabited by millions of interacting organisms [40]. Microbes (bacteria, fungal decomposers, mycorrhizal fungi) are the foundation of the soil food web supporting key processes such as photosynthesis and organic matter decomposition [41, 42]. Earthworms are also critical decomposers in this food web; by decomposing organic matter, they release essential nutrients for plants and create biopores that enhance soil structure and water infiltration. Much less studied, predators such as protists, nematodes, mites, and springtails, also support soil food webs. Unlike animal–plant (e.g., bee–plants; birds–plants) ecological networks, soil networks are extremely complex, and it is impossible to examine the simultaneous interactions of hundreds of thousands of nodes using current technology. In an attempt to better understand soil food webs, ecological networks generated from high-throughput sequencing data have increased in popularity in microbial ecology in the last decade [42–44]. Such potential interactions can be explored by examining co-existence patterns in soil organisms through “microbial co-occurrence networks” or “microbial association networks”, which are based on the natural correlations in the relative abundance or presence of soil taxa and genes. Of course, correlation does not always reflect causality. While these tools offer a great opportunity to better understand the complexity of microbial communities, they also raise new challenges related to the specific characteristics of soil datasets and the type of ecological questions that can be addressed. Differences in soil heterogeneity [45] and physico-chemical conditions may make it difficult to account for inter- and intra-sample variability.

Soil ecological networks, which also have their limitations, have helped us to advance our understanding of the major global patterns in soil biodiversity and function. For example, networks have improved our understanding of the ecological preferences of individual soil taxa. Soil taxa within ecological networks are known to group themselves into “ecological clusters” or “modules” composed of taxa that are highly correlated. These modules allow us to explore how a large group of individual taxa is correlated with their environment, providing important insights into their environmental preferences. Understanding the ecological traits of microbial phylotypes by identifying ecological modules could increase our ability to predict how soil organisms respond to changes in precipitation or soil pH [46–50]. Moreover, network modules allow us to better forecast the future of soil biodiversity under global change. For example, recent work suggests that increases in temperature and aridity such as those forecasted for the next few decades in drylands could potentially lead to drastic changes in the community composition of functionally important bivariate networks within soil food webs [49]. Furthermore, soil ecological networks allow us to better understand how the biodiversity of soil taxa correlates with ecosystem function. For example, this approach suggested that the richness of taxa (i.e., number of species) within certain ecological modules can be particularly important in supporting multifunctionality [9]. We know that the diversity of “hub taxa” (i.e., highly connected within and/or between ecological modules) is fundamental for supporting ecosystem function [9]. More recently, the exploration of network motifs [51], encompassing associations among taxa triads, has allowed us to better understand how soil organisms are associated within soil networks. Examining soil ecological networks involving multiple trophic levels, from microbes such as bacteria and fungi to soil micro and macrofauna (e.g., earthworms), across contrasting regions of the world, has revealed the importance of positive associations among organisms and their role in enhancing soil biodiversity and stability in the face of global change [9,50,51]. Functional gene co-occurrence networks and machine learning classification analyses can further help us to identify specialized metabolic functions, e.g., “nitrogen metabolism” and “phosphonate and phosphinate metabolism,” and their associations with soil organisms such as *Actinobacteria* and *Proteobacteria* (e.g., *Nitrospira* and *Gemmatimonas* spp*.)* [52]. Finally, network analyses are important to infer associations between soil organisms within the food web, including, for example, predator–prey (e.g., protists–bacteria [49]) and pathogen–host (e.g., virus–bacteria [53]) relationships.

## Next-generation studies of soil microbial diversity and function

Future studies of soil biodiversity and ecosystem functions should look for a deeper mechanistic understanding of how soil microbes interact at gene and chemical levels in order to advance our knowledge of how soil biodiversity-function relationships develop. Chemicals are necessary for communication for plant-to-microbe and microbe-to-microbe interactions. Tapping into this chemical communication is essential to understand how biodiversity–function relationships emerge and are maintained. Recent work suggests that local adaptation of plant host immune systems (e.g., a crop species) is influenced by microbial-associated molecular patterns (MAMPs) that selectively shape the evolution of their pattern recognition receptors (PRRs), ultimately promoting fitness [54]. Exposure to a wide variety of MAMPs from diverse microbial communities helps plants remember different molecular structures, speeding up the process of identifying new, harmful pathogens [55]. The availability of diverse sets of PRRs defines immunity-microbial homeostasis and likely serves an essential function in shaping beneficial plant-microbiota combinations. Similarly, it is becoming more widely accepted that the primary pathway by which plant species diversity improves microbial-mediated ecosystem functions, including soil carbon storage, is through root exudates (fluids released by roots into the soil) [56–59]. Although root exudates have been connected to microbial biomass and fungal:bacterial ratios in mixed plant communities, the mechanism by which chemical diversity of metabolites in root exudates influence soil biodiversity remains poorly understood [57]. Interestingly, the experimental addition of root exudate cocktails to plant microcosms has been shown to overwhelm plant diversity impacts on soil microbiota [60]. Similarly, little is known about how microbes communicate, for example, through signal molecules and quorum sensing skills. More information is needed on how chemical signaling regulates the diversity–function links between plants and microbes at the ecosystem level.

In addition to chemistry investigations, “-omics” approaches including metagenomics, transcriptomics, proteomics, and metabolomics still need to provide a deeper contribution to our understanding of soil microbial biodiversity–ecosystem functioning (BEF) relationships. This is particularly valuable for understanding ecosystem processes such as nutrient cycling and contaminant degradation. For example, metagenomics can evaluate several functions simultaneously, avoiding overestimating functional redundancy and recognizing the value of multifunctionality [61]. Adopting a functional gene-centric approach will provide a better understanding of the soil microbial BEF relationships than a taxonomic-based approach. A recent meta-analysis using metagenomics data showed that while biodiversity loss caused a decrease in taxonomic species by 72%, changes in the relative abundance of diverse functional categories were limited [62]. Metagenomics can also help us to construct high-quality metagenomics assembled genomes that provide an essential resource to help us unravel the yet-unknown microbial diversity environments and their functional potential [63, 64]. Metagenomics offers a connection between the variety of different microbial species present in an environmental sample (biodiversity) and the range of functional capabilities encoded within their collective genetic material (metagenome). We postulate that in the future, a reduction in the sequencing costs combined with improved computational tools for assembly and annotation of environmental genes and genomes will permit a greater use of metagenomics to explore BEF relationships.

In addition, genome-scale metabolic models (GEMs), which provide a mechanistic understanding of how individual genes and molecules collectively explain cell- and ecosystem-level fluxes, is an emerging approach to predict process and system dynamics based on genomic and phenotypic properties of microbial communities [65–67]. While GEMs were initially developed to study individual microbial species, these approaches have now been extended to microbial communities, enabling *in silico* experiments in which multiple species can compete or synergize based on molecular exchanges [68–70]. A remarkable aspect of these models is that they can translate genomic information, particularly the list of reactions in each organism, into predictions of metabolic phenotypes, including growth capability, intracellular reaction rates, uptake/secretion fluxes, and processes. These simulation methods are almost ripe for modeling complex microbial communities to better understand how different species and their interactions affect ecosystem functions. For example, GEMs were used to form a network of rhizosphere microbiome communal trophic dependencies that play crucial role in biodiversity–function relationship [69]. GEMs can also be used to design and parameterize multi-scale agent-based models integrating metabolic/kinetic models, spatiotemporal dynamics, and biogeochemical models informed by machine learning [71, 72].

## Soil biodiversity in the context of multiple global change stressors

**Fig 2:**
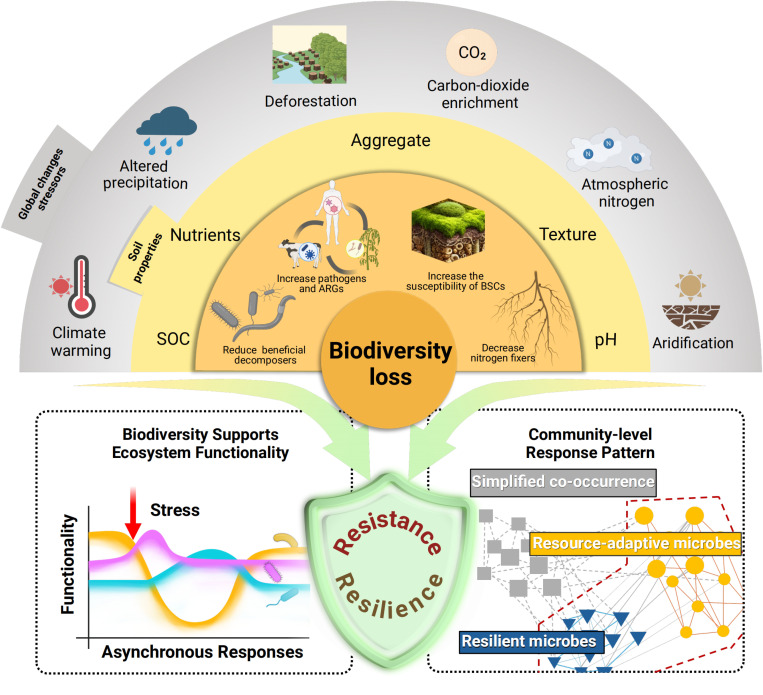
Adapting to multiple global change stressors. Multiple global change stressors are impacting the biodiversity and function of soils, with consequences for the resilience and resistance of ecosystems. An increase in the number of multiple global change factors, including enhanced CO_2_, fertilization, warming, and drought (to name a few), can result, for example, in important reductions in the resistance of key ecosystem services driven by soil organisms including soil carbon stocks, soil fertility, soil microbial habitat, and soil organic matter decomposition to on-going global changes [73].

Human-induced and natural global change factors such as climate warming, carbon-dioxide enrichment, altered precipitation, acidification, nitrogen deposition, land-use change, and combinations thereof, pose an existential threat to the planet’s biodiversity (Fig 2) [73–78]. It is therefore critical to assess how these drivers might influence the effects of soil biodiversity on ecosystem functions and, ultimately, on ecosystem sustainability [74–76]. However, the biodiversity of soil organisms is not only highly vulnerable to global environmental changes but also land use intensification processes such as urbanization, deforestation, grazing, and agricultural land uses. For example, urban greenspaces such as parks and gardens support homogenized microbiomes characterized by greater proportions of plant, animal, and human pathogens, and antibiotic-resistance genes [75]. Moreover, deforestation processes reducing 10 billion trees each year are known to reduce soil carbon stocks and beneficial decomposer organisms, while promoting the proportion of soil-borne pathogens [74]. Another major factor reshaping soil biodiversity is agricultural practices, particularly fertilization and soil disturbance. We know, for example, that long-term fertilization can eliminate critical organisms such as nitrogen fixers, which are out-competed by other organisms when nitrogen is added to the soil. Overgrazing by livestock can alter the number of species and community composition of soil microbes by changing competitive networks [76]. Similarly, biocrusts are threatened by human-induced land use changes such as disturbance by motor vehicles, humans, and livestock, and fire. Trampling and disturbance destroy the external structure of crusts while fire destroys the mucilaginous material produced by the fungal and cyanobacterial components of lichens, making them susceptible to breakdown and removal. Land use changes also affect water availability, which is known to result in critical environmental thresholds; i.e., under a given level of water availability, small changes in water availability can drastically alter soil biodiversity and function. This is particularly important in drylands where aridification reduces the diversity of soil organisms and ecosystem multifunctionality, which is exacerbated by global warming. For example, warming is known to promote the proportion of soil-borne fungal pathogens [43], while reducing the proportion of beneficial fungal decomposers and soil organic carbon stocks. Climate is also a major driver of the abundance and diversity of soil micro and macrofauna including nematodes, earthworms, and mites [11,12,78].

All of these individual global change processes are fundamental in explaining soil biodiversity and function in the Anthropocene. However, we should not forget that soils are not only subjected to individual processes but to a myriad of multiple and simultaneous environmental natural and anthropogenic stressors. Recent global-scale observational and experimental research suggests that as the number of stressors increases, both soil biodiversity and function decline [77]. Similarly, an increasing number of stressors limits the capacity of ecosystems to resist global change impacts [75]. Much less is known about the impacts of multiple global change stressors on the abundance, community composition and diversity of macro- and microfauna including earthworms or mites and springtails or nematodes. New research should focus on better understanding how and why multiple environmental stressors interact and affect ecosystems in a rapidly changing world.

## Resilience and global change adaptation of soil biodiversity and function

Advancing our understanding of the capacity of soil biodiversity, including microbes, micro- and macrofauna, to remain stable in response to global change is fundamental for supporting ecosystem functions, as microbes regulate the response of soil function to global change [79]. Previous studies have demonstrated that microbes regulate the responses of multifunctionality to warming, drought, and fertilization in soils from drylands worldwide [47]. Moreover, biocrusts can mitigate the impacts of aridification on soil biodiversity and function [80]. Yet, the capacity of microbes to support function under global change may depend on their capacity to withstand stress. For example, in 2022, researchers investigated the effects of soil microbial diversity on soil functions and properties when faced with an increasing number of simultaneous global change factors in experimental microcosms [81]. Higher soil microbial diversity had a positive effect on soil functions and properties when either a few or no global change factors were applied, but this positive effect was eliminated by the co-occurrence of numerous global change factors. Moreover, we need a better understanding of how different groups of organisms, including microbes, macro- and microfauna co-existing in soils influence the capacity of soil biodiversity to support function under global change. For example, plant functional groups have recently been shown to modulate the response of soil function–microbial diversity relationship under climate change [82]. Despite the fundamental importance of soil microbes, little is known about how their stability is affected in response to multiple global change stressors.

In general, soil microbes, micro- and macrofauna seem to be largely vulnerable to global change, with dominant taxa being less sensitive to global changes compared to rarer taxa [83]. The temporal stability of soil biodiversity under global change depends on the resilience (the community recovery process to an alternative stable state) and the resistance (the community remains unchanged in response to a disturbance) [84] of this soil biodiversity to multiple environmental stressors. For soils, it is well known that stability depends on biodiversity and the structure of the soil food web [85]. In a diverse ecosystem, not all organisms are likely to be impacted by a disturbance regime in the same way. In other words, the more diverse a community’s molecular makeup, the more resilient and adaptable it can be to environmental changes, as this community can support a wider range of biological processes such as resource utilization, decomposition, and nutrient cycling. In this respect, asynchronous soil microbial communities have been demonstrated to play a critical role in supporting ecosystem stability. Moreover, the stability of soil biodiversity is driven by physico-chemical soil properties and legacies. For example, a recent study suggests that the response of soil microbial activities to warming is driven by previous global change legacies affecting those soils [86]. Similarly, land management influences the responses of dominant soil microbial taxa to droughts across grasslands at a regional scale [82].

How soil biodiversity will adapt to ongoing global change remains poorly understood. We would expect that some microbes are highly resistant to extreme conditions [87], yet this is often not the case. Survival in increasingly stressful environments requires specialized mechanisms of adaptation, and along aridity gradients, soil communities are progressively enriched with genes related to this stress tolerance, harboring, for example, smaller and simplified genomes and reduced community stability and interactions [88]. Consequently, increases in the incidence of stress tolerance (e.g., sporulation, motility, dormancy), resource scavenging traits, and simplified co-occurrence patterns, are likely outcomes of community-level adaptations to increasing water depletion (aridification) driven by global change. Conversely, wetter Arctic soils may be particularly vulnerable to climate change as biological and chemical processes in cold ecosystems are more sensitive to temperature fluctuations [89]. More recently, it has been emphasized that soil biodiversity increases with global warming in cold and very cold environments, and thus global warming is expected to have a positive impact on biodiversity in these regions [90].

## Toward the conservation of soil biodiversity

Soil biodiversity is key for function, but is highly vulnerable to global change [91]. Therefore, it is critical that we learn how to protect soil biodiversity for future generations. Unfortunately, soil biodiversity does not follow the same general patterns as the biodiversity of plants [92, 93]; this limits our capacity to protect soil microbes by protecting plants. The first step toward soil biodiversity conservation is to identify the major global patterns and environmental preferences of soil biodiversity. Fortunately, we have more than 20 years of literature characterizing the environmental factors associated with soil biodiversity [12–14]. We know, for example, that soil pH and plant cover (i.e., carbon inputs) are the two most important factors controlling overall soil biodiversity, including bacteria, fungi, protists, and invertebrates as soil develops from centuries to millions of years old [91]. In particular, in highly productive ecosystems such as warm and wet ecosystems, changes in soil pH (acidification) as soil develops are known to control changes in soil biodiversity. These changes normally follow a hump-shaped relationship with soil pH [12]. However, in ecosystems of low productivity such as arid and cold environments, pH changes very little as soil develops, and the development of plant cover is positively associated with soil biodiversity as the ecosystem develops [94]. The importance of factors such as soil pH as controllers of microbial diversity has been known for decades [12]. Moreover, these major patterns are now also contextualized within ecosystem models (e.g., via structural equation modeling). For example, in drylands, increased aridity leads to a reduction in plant cover, soil carbon, and soil microbial diversity, with pH playing a minimal role in shaping soil biodiversity [94].

**Fig 3:**
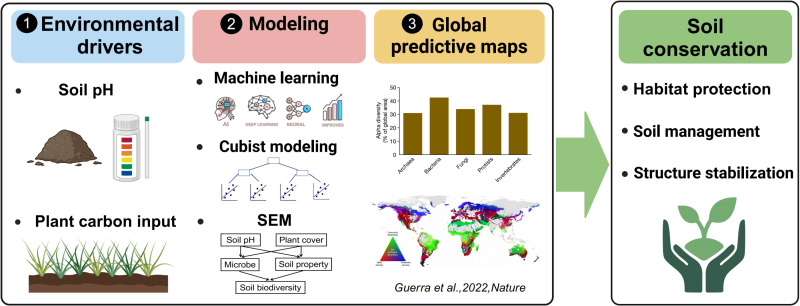
Toward conservation of soil biodiversity. Environmental models can help us predict the major drivers of biodiversity and function, which is critical to predict the current and future distributions of soils. Machine learning (e.g., Random Forest) has proven to be very useful when identifying the main environmental factors associated with soil biodiversity, especially when dealing with non-parametric data [e.g., 95]. Random Forest can also be used to identify soil taxa indicators of environmental conditions such as management types. Both Random Forest and Cubist are also key for mapping the global distribution of soil organisms [e.g., 13, 95]. Finally, Structural Equation Modeling (SEMs) can provide significant insights into the direct and indirect environmental factors associated with soil biodiversity [e.g., 35,49,77].

Understanding the major environmental factors affecting the soil microbiome has allowed scientists to develop the first global predictive maps of soil biodiversity using machine learning and cubist modeling (Fig 3). This includes the first global map of dominant bacteria, fungi, nematodes, rotifers, and earthworms [11,12,95–99]. These maps predict the regions of the planet supporting the greatest soil biodiversity. These types of maps have existed for decades for plants and animals but were clearly lacking for soil organisms. Similarly, the first global map of the hotspots of soil biodiversity and ecosystem services appeared in 2022 [98], highlighting cold forests as major reservoirs of ecosystem services such as carbon sequestration, temperate ecosystems as major hotspots of soil species richness, and tropical environments as major hotspots of soil taxa dissimilarity. These maps are a step forward toward the conservation of our soils. Unfortunately, they also highlight that around 90% of all global hotspots in soil biodiversity and ecosystem services remain unprotected, outside natural and national parks [98]. Much less is known, however, about the global distribution and vulnerabilities of soil viruses [20], with some important research recently conducted [20,100]. For instance, [20] put together a global database on soil metagenomes and showed that soil texture and moisture were important predictors of the distribution of soil-borne virus diversity finding higher diversity in humid and subhumid regions. Moreover, [100,101] discussed the importance of including viruses in soil food webs in order to better understand their impact on soil function and health. Developing national strategies to protect soil biodiversity is, therefore, urgently needed. This includes the need to understand how well the current reserve system is protecting soil biodiversity, and to develop national surveys and national and international governmentally supported soil biodiversity databases. This will help us to prevent the invasion of potential soil aliens, an issue that remains poorly understood and underappreciated compared with other systems such as marine systems. The use of artificial intelligence could play a critical role in soil biodiversity databases by providing, for example, soil fauna identification and linking citizen science (e.g., real-time mobile apps such as PlantNet or iNaturalist) to the conservation of soil biodiversity.

## Conclusions and future perspectives

Our knowledge of soil biodiversity and function has vastly increased over the last 20 years. Soil biodiversity is essential for supporting ecosystem multifunctionality. However, soil biodiversity is also highly vulnerable to multiple global change stressors. Because of this, we urgently need to learn how to conserve this biodiversity and function for future generations. Our current understanding of the environmental factors controlling soil biodiversity has allowed us, for the first time in history, to create global maps of soil biodiversity, and to identify the major hotspots of soil biodiversity and ecosystem services. Yet, we are still far from being able to efficiently conserve soil biodiversity, with most hotspots of soil biodiversity and ecosystem services remaining largely unprotected.

**Fig 4:**
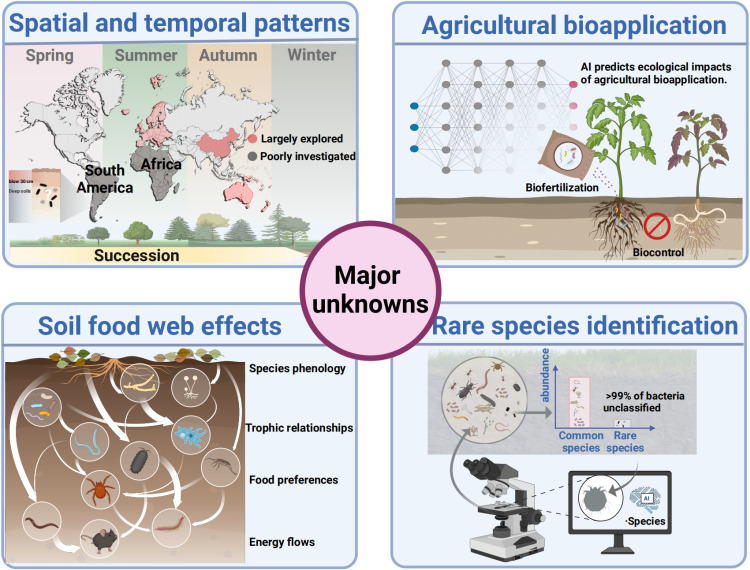
Major unknowns in soil biodiversity. This figure includes some examples on the major unknowns limiting our understanding on soil biodiversity. Some of these unknowns include: (1) The existence of major global-scale spatial coldspots, with data availability in Africa and South America still largely unexplored; (2) Seasonal patterns in soil biodiversity; (3) A poor understanding on how to harness synthetic microbial communities (SynComs) to promote key ecosystem services such as food production and carbon sequestration; (4) Knowledge about how microbes interact with each other, and with other organisms within the soil food web; and (5) lack of taxonomic information describing most soil organisms, especially rare taxa, which have a large impact on our capacity to conserve these organisms for the next generations.

It is important to address the major knowledge gaps and where research needs to be directed to promote soil conservation over the next few decades (Fig 4). Some of these include the need to fill major global gaps in soil biodiversity surveys. While some locations of the planet are largely explored including Europe (e.g., LUCAS survey), China (i.e., the China Microbiome Initiative [102]), and Australia (The Australian Microbiome initiative), others such as South America or Africa remain relatively poorly studied. A better resolution in global databases is critical for improving our understanding of the status of conservation of soil biodiversity and function. Of major concern is the lack of information on the taxonomy of soil organisms. If a soil organism is unknown, it cannot be protected. For example, it is commonly estimated that >99% of all bacterial species remain unclassified. Even for the most dominant bacterial taxa, we know only a small percentage of their taxonomy. We do not know who they are or what they are doing at the individual level. Taxonomic limitations are also crucial for tracking the entrance of invasive microbial, macro- and microfauna organisms in our soils. Temporal monitoring is needed to better understand the conservation of any organisms, yet we are lacking more efforts in producing these types of data. We know that soil biodiversity changes over seasons, succession, and ecosystem development. Therefore, temporal surveys are needed to determine if soil taxa are in decline. The lack of taxonomic data on soil organisms limits our capacity to monitor and protect these organisms for the next generations. A lesser-known consequence of our limited understanding is the potential loss of hundreds of microbial species thriving within soil organisms such as earthworms [103], whose own extinction would trigger a cascade of secondary extinctions within their gut microbiomes.

Finally, while most efforts focus on common taxa and topsoil biodiversity, we have a very poor understanding of the biodiversity of rarer taxa and the profile of life deep in the soil because most of our efforts have focused on the immediate surface layers. Other major knowledge gaps include our lack of knowledge on soil species phenology, trophic relationships (brown food webs) and food preferences, or learning more about energy flows in trophic levels and interaction (rather than correlation) mechanisms in explaining soil networks and functions. These knowledge gaps make it difficult to conserve soil biota, with the International Union for Conservation of Nature Red List of Threatened Species lacking a more complete list of threatened soil organisms. Conserving soil biodiversity is paramount for ensuring food security and maintaining interactions among crop species and soil taxa that are necessary for sustainable farming practices. However, preserving soil biodiversity is also crucial for maintaining a wide range of ecosystem services, including those related to One Health, which connects the health of humans, animals, and the environment.

## Abbreviations

**:**
BEF: biodiversity–ecosystem functioning
GEMs: genome-scale metabolic models
MAMPs: microbial-associated molecular patterns
PRRs: pattern recognition receptors
